# Bariatric Surgery Efficiency, Safety and Health Outcomes in Government Versus Privately Funded Hospitals

**DOI:** 10.1007/s11695-023-06489-3

**Published:** 2023-02-16

**Authors:** Chiara Chadwick, Paul R. Burton, Dianne Brown, Jennifer F. Holland, Angus Campbell, Jenifer Cottrell, Andrew D. MacCormick, Ian Caterson, Wendy A. Brown

**Affiliations:** 1 Department of Surgery, Central Clinical School, Alfred Health, Monash University, Level 6, The Alfred Centre, 99 Commercial Road, Melbourne, 3004 Australia; 2 Oesophago-Gastric and Bariatric Unit, Alfred Health, Melbourne, Victoria 3004 Australia; 3 School of Public Health and Preventive Medicine, Bariatric Surgery Registry, Monash University, Melbourne, 3004 Australia; 4 Department of Surgery, University of Auckland, Auckland, Aotearoa New Zealand; 5 Boden Initiative, Charles Perkins Centre, University of Sydney, New South Wales 2006 Sydney, Australia; 6 Department of Endocrinology, Royal Prince Alfred Hospital, Sydney, 2050 Australia

**Keywords:** Obesity, Bariatric surgery, Outcomes, Efficiency, Safety

## Abstract

**Purpose:**

This study aims to determine if the hospital efficiency, safety and health outcomes are equal in patients who receive bariatric surgery in government-funded hospitals (GFH) versus privately funded hospitals (PFH).

**Materials and Methods:**

This is a retrospective observational study of prospectively maintained data from the Australia and New Zealand Bariatric Surgery Registry of 14,862 procedures (2134 GFH and 12,728 PFH) from 33 hospitals (8 GFH and 25 PFH) performed in Victoria, Australia, between January 1st, 2015, and December 31st, 2020. Outcome measures included the difference in efficacy (weight loss, diabetes remission), safety (defined adverse event and complications) and efficiency (hospital length of stay) between the two health systems.

**Results:**

GFH treated a higher risk patient group who were older by a mean (SD) 2.4 years (0.27), *P* < 0.001; had a mean 9.0 kg (0.6) greater weight at time of surgery, *P* < 0.001; and a higher prevalence of diabetes at day of surgery OR = 2.57 (CI_95%_2.29–2.89), *P* < 0.001. Despite these baseline differences, both GFH and PFH yielded near identical remission of diabetes which was stable up to 4 years post-operatively (57%). There was no statistically significant difference in defined adverse events between the GFH and PFH (OR = 1.24 (CI_95%_ 0.93–1.67), *P* = 0.14). Both healthcare settings demonstrated that similar covariates affect length of stay (LOS) (diabetes, conversion bariatric procedures and defined adverse event); however, these covariates had a greater effect on LOS in GFH compared to PFH.

**Conclusions:**

Bariatric surgery performed in GFH and PFH yields comparable health outcomes (metabolic and weight loss) and safety. There was a small but statistically significant increased LOS following bariatric surgery in GFH.

**Graphical Abstract:**

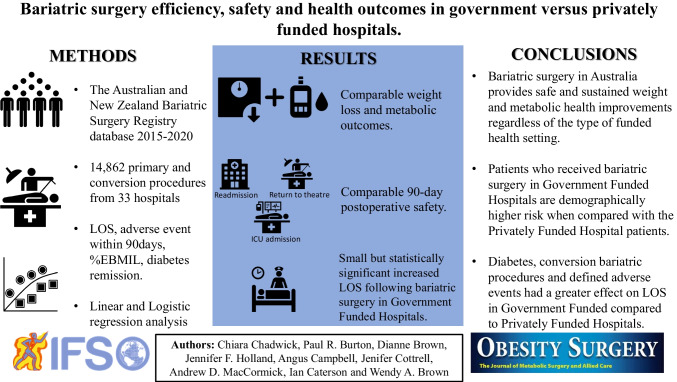

## Background

Overweight and obesity are the second highest contributors of total disease burden in Australia, responsible for 8.4% of total disability-adjusted life years (DALY) [1]. There is a similar situation in other Organisation for Economic Co-operation and Development (OECD) countries [2]. Individuals with obesity are more frequent users of healthcare services; many diseases associated with obesity will remit with weight loss; therefore, effective treatments for obesity are clinically, economically and socially important. People with obesity are more likely to be of lower socio-economic means when compared to people with BMI below 30 kg/m^2^, so equitable pathways to enable access to effective treatments for obesity are required [3]*.*

Bariatric surgery is widely accepted as an effective and durable treatment for obesity. As with the international experience, the demand for bariatric surgery in Australia is steadily increasing [4, 5]. From 2015 to 2019, the number of bariatric procedures performed per annum increased from 15,290 (9.7 hospital separations per 100,000 Australians) to 21,315 [6, 7]. Recently published forecasting models have estimated that a ninefold increase in demand for bariatric surgery in Australia is predicted within the next decade [8].

Access to bariatric surgery in Australia is predominantly via the private sector where individuals either pay directly for their surgery or access private health insurance [6]. For those unable to afford private care, there is limited access to bariatric surgery in government-funded hospitals (GFH) which is provided at no cost to the individual receiving care [3]. Funding for GFH comes from government tax revenue, meaning GFH have finite resources. Any procedure offered in GFH is required to have a strong evidence base not only for health benefit, but also, ideally, cost benefit for the community who are the payer.

There is currently a paucity of data confirming the efficacy, safety and cost efficacy related to bariatric surgery in GFH. Most available evidence has been generated in the private sector with information from GFH only available from a few series [9, 10].

The Australia and New Zealand Bariatric Surgery Registry (ANZBSR) is a clinical quality collaborative which was established in 2009 and since 2012 has collected data pertaining to the safety and efficacy of bariatric surgery in over 120,000 individuals with obesity [6]. This contemporaneously maintained clinical quality registry currently includes 75% of all bariatric procedures performed in Australia annually^6^. Utilising data from the ANZBSR, the aim of this study was to determine if health outcomes, procedural safety and hospital efficiency (as reflected in hospital LOS) is equivalent for patients who have undergone bariatric surgery in GFH and privately funded hospital (PFH) settings.

## Method

We have performed a retrospective observational analysis of prospectively maintained data from the Australian and New Zealand Bariatric Surgery Registry (clinicaltrials.gov ID: NCT03441451). Ethics approval was obtained from The Alfred Hospital Human Research Ethics Committee (Ref 75/20).

### Cohort Selection

**Fig. 1:**
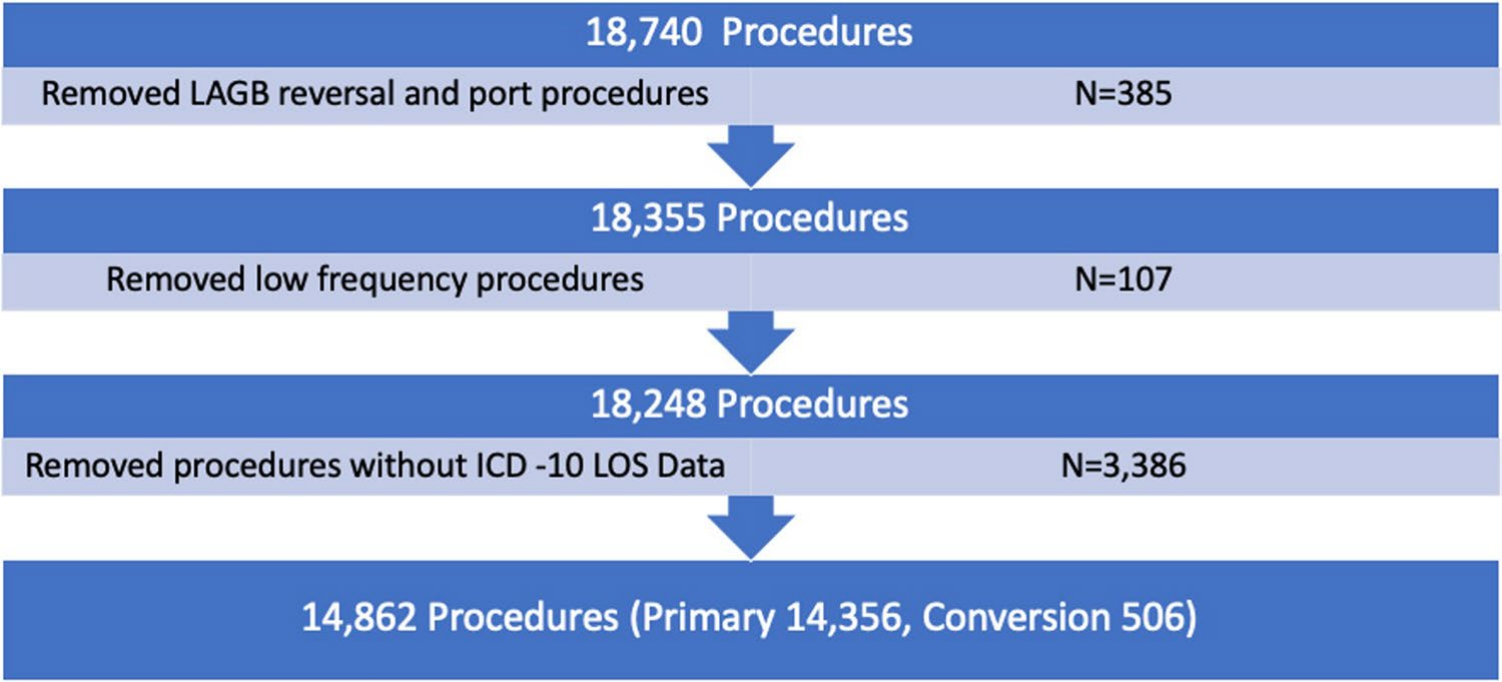
Cohort selection

This study included deidentified data of bariatric patients who had their primary procedure performed in GFH and PFH in Victoria, Australia, between January 1st, 2015 and December 31st, 2020, and were enrolled with the ANZBSR and excluded participants under 18 years of age and who identified as neither male nor female (*n* = 23). Procedures were also excluded if their length of hospital stay data was missing (Fig. 1). In addition, uncommonly performed procedures were excluded as their incidence was too low for subgroup statistical analysis. These procedures represented 0.57% of bariatric procedures performed.

### Outcome Variables

Patient covariates included age (years) at time of procedure, sex, weight (kg) and BMI (kg/m^2^) at day of surgery, diabetic status and the type of diabetic therapy an individual with diabetes received. Diabetic status was reported as “yes”, “no” or “unknown/not disclosed”. This was recorded at time of operation and at annual follow-up. Therapy for diabetes was reported at date of surgery and at annual follow-up and categorised as diet/exercise, non-insulin monotherapy, non-insulin polytherapy, insulin and unknown/not disclosed. Health outcomes between the two health care settings were compared using percentage excess BMI loss (%EBMIL) and remission of diabetes. These outcomes were reported annually following bariatric surgery.

Procedure covariates included procedure type and operation status. Procedure type was defined as the specific bariatric procedure performed being sleeve gastrectomy (SG), Roux-en-Y gastric bypass (RYGB), one anastomosis gastric bypass (OAGB) and laparoscopically inserted adjustable gastric band (LAGB). Operation status defined whether a bariatric operation was a primary or conversion procedure. A primary bariatric procedure was the first bariatric procedure an individual had received. A conversion bariatric procedure was a procedure that anatomically changed the index bariatric procedure to a different type of bariatric procedure [11]. Stand-alone LAGB reversal procedures and LAGB port revision procedures were excluded as these did not satisfy the definition of a conversion bariatric procedure [11].

Healthcare efficiency and safety following primary and conversion bariatric surgery were compared between GFH and PFH using hospital length of stay and the occurrence of defined adverse events. Hospital length of stay is the number of hospital bed days recorded for the bariatric procedure-related admission. In Australian hospitals, principal diagnoses are classified according to the International Statistical Classification of Diseases and Related Health Problems, Tenth Revision, Australian Modification (ICD-10-AM) [12]. Hospital-admitted patient data collected by the ANZBSR included episodes of care associated with procedures for bariatric surgery. The ANZBSR records defined adverse events as unplanned return to theatre, unplanned ICU admission or unplanned readmission to hospital occurring within 90 days of the bariatric procedure. Specific complications are also reported, and in this study, these were haemorrhage, reflux/dysphagia, wound associated, leak, stricture/stenosis, torsion, internal hernia, abdominal pain, bowel obstruction, malnutrition, thromboembolism and LAGB associated.

### Statistical Analysis

Data were approximately normally distributed due to the large sample size [13]. Descriptive statistics are represented as percentages for categorical data variables. Continuous data variables are described as mean and standard deviation (SD) or mean and 95% confidence intervals (CI_95%_). The *t* test was performed to determine the significance of difference in health outcomes between the two healthcare settings. Logistic regression was used to determine the difference in odds for occurrence of defined adverse events in GFH compared to PFH. ANOVA was performed to determine the significance of intergroup differences in LOS and procedure type, primary and conversion procedures and patient demographic variables. Linear regression analysis determined the relative change to LOS associated with patient demographic variables, primary versus conversion procedures, procedure type and defined adverse event. A *P* value of less than 0.05 was considered significant. All statistical analyses were performed using IBM SPSS version 28 (SPSS Inc., Chicago, IL, USA).

## Results

### Demographics

This study included 14,862 procedures (2134 GFH and 12,728 PFH) from 33 hospitals (8 GFH and 25 PFH); 95.5% were primary bariatric procedures. The population was predominantly female (79.5%). Participants who received bariatric surgery in GFH were a mean (SD) 2.4 years (0.27) older than individuals in PFH settings, *P* < 0.001 (Table 1).

**Table 1:** Patient demographics between healthcare settings

Demographic	GFH	Privately funded	Mean difference	*P* value
Procedures *N*	2134 (14.3%)	12,728 (85.7%)	-	-
Gender				0.49
Male	449 (21%)	2596 (20.4%)	-	
Female	1685 (79%)	10,132 (79.6%)	-	
Operation status				0.003
Primary procedure	2038 (95.5%)	12,320 (96.8%)	-	
Conversion procedure	96 (4.5%)	408 (3.2%)	-	
Diabetes (yes)				< 0.001
Primary procedure	507 (25.8%)	1288 (12.0%)	-	
Conversion procedure	21 (25.6%)	21 (6.9%)	-	
Age	44.1 years (11.2)	41.7 years (11.5)	2.4 years (0.2)	< 0.001
Primary procedure				
Mean (SD)	44.1 years (11.3)	41.7 years (11.6)	2.4 years (0.3)	< 0.001
Range	18–84 years	18–76 years		
Conversion procedure				
Mean (SD)	45.2 years (10.3)	42.5 years (10.7)	2.6 years (1.2)	0.028
Range	24–70 years	20–74 years		
Weight at operation	128.6 kg (26.1)	119.6 kg (24.2)	9.04 kg (0.60)	< 0.001
Primary procedure				
Mean (SD)	129.3 kg (25.7)	120.1 kg (23.9)	9.2 kg (0.6)	< 0.001
Range	73–272 kg	65–300 kg		
Conversion procedure				
Mean (SD)	113.8 kg (28.2)	106.9 kg	6.92 kg (3.1)	0.028
Range	61–190 kg	60–200 kg		
BMI at operation	46.2 kg/m^2^ (8.1)	42.4 kg/m^2^ (7.2)	3.87 kg/m^2^ (0.18)	< 0.001
Primary procedure				
Mean (SD)	46.4 kg/m^2^ (7.9)	42.5 kg/m^2^ (7.1)	3.9 kg/m^2^ (0.1)	< 0.001
Range	31–92 kg/m^2^	18–92 kg/m^2^		
Conversion procedure				
Mean (SD)	41.1 kg/m^2^ (9.0)	37.9 kg/m^2^ (8.3)	3.2 kg/m^2^ (3.2)	0.001
Range	24–66 kg/m^2^	21–69 kg/m^2^		

^*^ *P* value derived from Pearson’s chi-squared test for categorical variables and Student’s *t* test for continuous variables

**Fig. 2:**
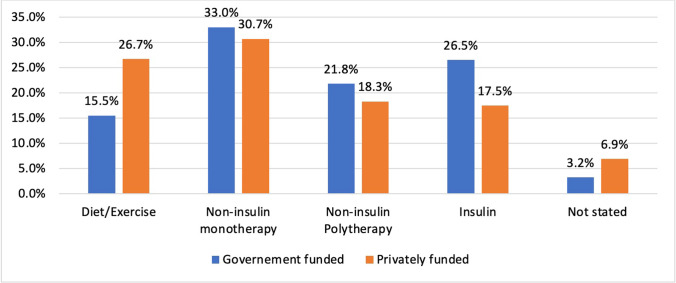
Management of diabetes at time primary procedure

Patients operated on in a GFH had a significantly greater mean weight and BMI at day of surgery compared to PFH with a mean difference of 9.04 kg (0.60), *P* < 0.001, and 3.87 kg/m2 (0.18), *P* < 0.001. One patient had a BMI of 18 kg/m2 at time of conversion surgery from sleeve gastrectomy to gastric bypass following excessive weight loss secondary to sleeve stenosis. GFH patients were more likely to have diabetes at time of surgery (OR = 2.57 (CI_95%_2.29–2.89), *P* < 0.001). Patients operated on in GFH with diabetes had a significantly increased risk of requiring more than one oral hypoglycaemic agent (OR = 2.55 (CI_95%_ 1.45–4.48), *P* = 0.001) and insulin (OR = 3.24 (CI_95%_ 1.85–5.66), *P* < 0.001) compared to PFH (Fig. 2).

**Fig. 3:**
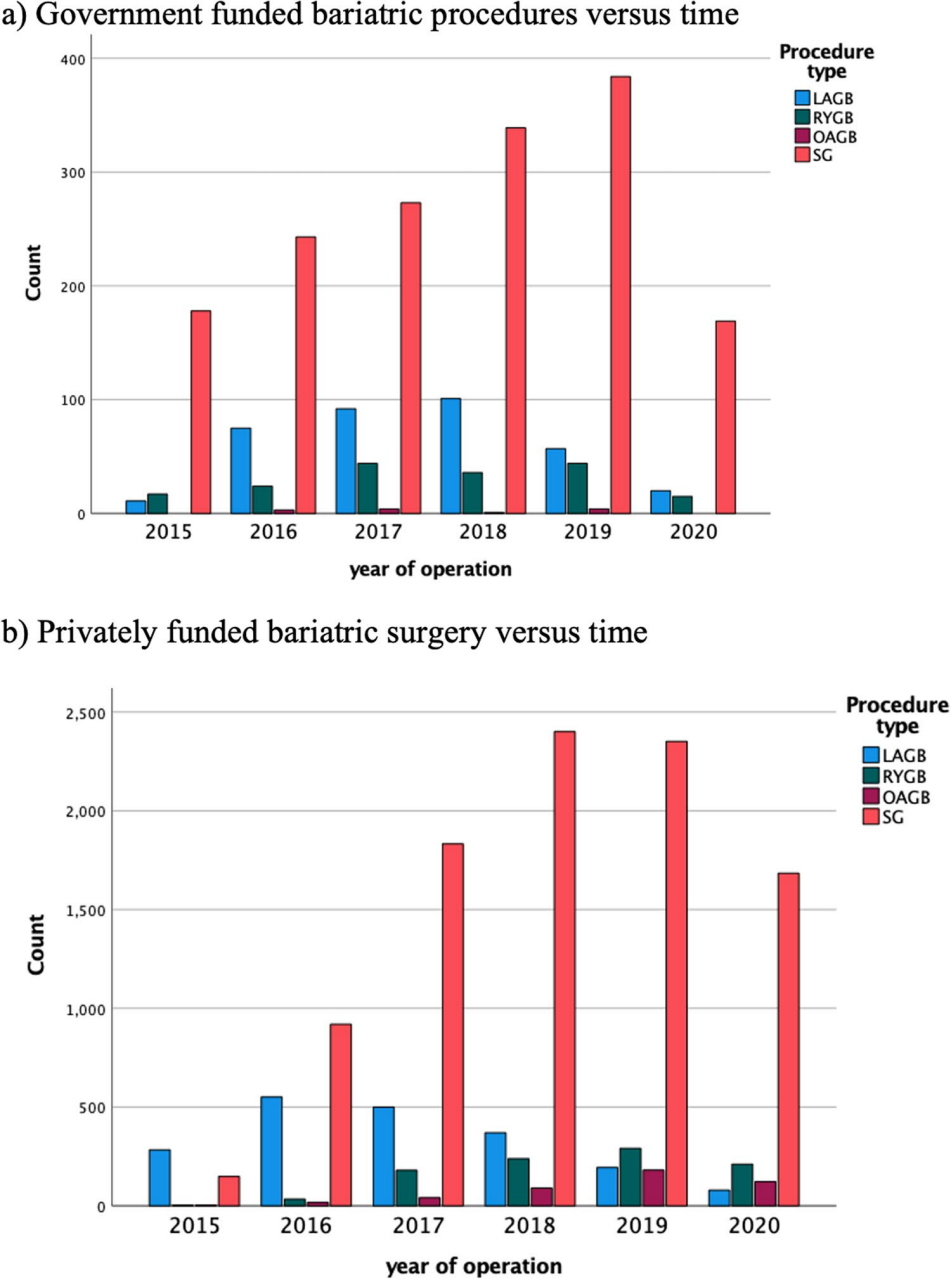
Procedure distribution between GFI and privately funded healthcare settings

Overall procedure type was similar across both healthcare settings (Fig. 3a and b). The SG was the most commonly performed bariatric procedure across both healthcare systems representing 76.3% of GFH and 74.8% of PFH procedures. The annual number of LAGB procedures has been declining across both healthcare settings from 47.7% (2015) to 20% (2017) and 4.3% (2020) of operations.

### Weight and Health Outcomes

**Fig. 4:**
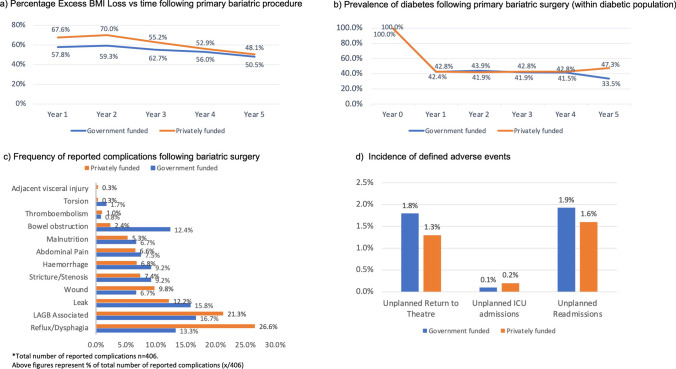
Health outcomes following bariatric surgery

Both healthcare settings demonstrated durable %EBMIL at 5 years post-bariatric surgery (Fig. 4a). PFH patients had a significantly higher mean %EBMIL in post-operative year 1 (9.78% (CI_95%_ 7.88–11.59), *P* < 0.001), year 2 (10.72% (CI_95_ 8.26–13.19), *P* < 0.001) and year 3 (7.55% (CI_95_ 4.14–10.96), *P* < 0.001) respectively. There was no statistically significant difference in mean %EBMIL between the two healthcare settings from year 4 (3.16% (CI_95%_ − 2.06–8.39), *P* = 0.23) to year 5 (2.34% (CI_95%_ − 6.56–11.24), *P* = 0.60).

Post-operative remission of diabetes was equivalent between bariatric procedures in GFH and PFH (Fig. 4b). Following an initial remission rate of 57%, in the first 12 months following bariatric surgery, the persistence of diabetes was stable from 2 to 4 years post-operatively. At 5 years, there was 4.5% recurrence of diabetes in individuals who had received bariatric surgery in PFH, whilst GFH patients experienced a further 8% remission in the prevalence of diabetes. However, this difference at year 5 was not statistically significant between the two healthcare settings, OR = 0.32 (CI_95%_ 0.03–3.08) *P* = 0.33.

### Procedural Safety

Defined adverse events occurring within 90 days of operation were reported in 0.5% of bariatric procedures in GFH and 2.1% of procedures in PFH. The incidence of defined adverse events is represented in Fig. 4d. Logistic regression analysis demonstrated the difference in the overall risk for the reported occurrence of a defined adverse event was not significant between GFH and PFH, OR = 1.24 (CI_95%_ 0.93–1.67), *P* = 0.14. When the different procedure types were considered in conjunction with hospital setting, there was no statistically significantly difference in risk of overall and specific defined adverse events for SG, RYGB and OAGB or LAGB.

There were 406 specific complications that were reported in our sample. These occurred in 5.6% of procedures performed in the GFH and 2.22% of procedures performed in PFH (Table 2). The incidence of each of the twelve complication types within the two hospital settings is demonstrated in Fig. 4c. This difference in overall complication risk in GFH compared to PFH was significant for RYGB (OR = 1.32 (CI_95%_ 1.0–1.74), *P* = 0.049) when the specific procedure type was considered. There was no statistically significant increased overall risk of complication in LAGB (OR = 1.02 (CI_95%_ 0.89–1.18), *P* = 0.75) nor for SG (OR = 0.91 (CI_95%_ 0.81–1.01), *P* = 0.06). The OAGB complication reporting was too low to determine procedure-specific risk of complication between the two healthcare settings (*n* = 12).

**Table 2:** Complications between GFH vs PFH bariatric procedures

	GFH		PFH		Difference between GFH vs PFH		
Complication	*N*	Prevalence	*N*	Prevalence	OR	SE	*P* value
LAGB associated	20	0.9%	61	0.5%	3.05	0.28	< 0.001
Reflux/Dysphagia	16	0.7%	76	0.6%	4.75	0.27	< 0.001
Wound	8	0.4%	28	0.2%	3.50	0.40	0.002
Leak	19	0.9%	35	0.3%	1.84	0.28	0.032
Abdominal Pain	9	0.4%	19	0.1%	2.11	0.41	0.065
Stricture/Stenosis	11	0.5%	21	0.2%	1.91	0.37	0.082
Bowel obstruction	13	0.6%	6	0.05%	0.46	0.49	0.117
Haemorrhage	11	0.5%	19	0.01%	1.73	0.38	0.150
Malnutrition	8	0.4%	15	0.1%	1.87	0.44	0.150
Thromboembolism	1	0.05%	3	0.02%	3.00	1.15	0.341
Internal hernia	2	0.1%	1	0.07%	0.50	1.22	0.571
Torsion	2	0.1%	1	0.07%	0.50	1.22	0.571

The difference in odds of a specific complication occurring after a bariatric procedure in GFH compared to PFH is presented in Table 2. Statistically significant increased odds were observed in LAGB-associated complications (OR = 3.05 (SE 0.258), *P* < 0.001), wound complications (OR = 3.5 (SE 0.40), *P* = 0.002), leak (OR = 1.84 (S.E. 0.285), *P* = 0.032) and reflux/dysphagia (OR = 4.75 (SE 0.275), *P* < 0.001) in participants who attended GFH compared to PFH.

### Hospital Length of Stay

**Fig. 5:**
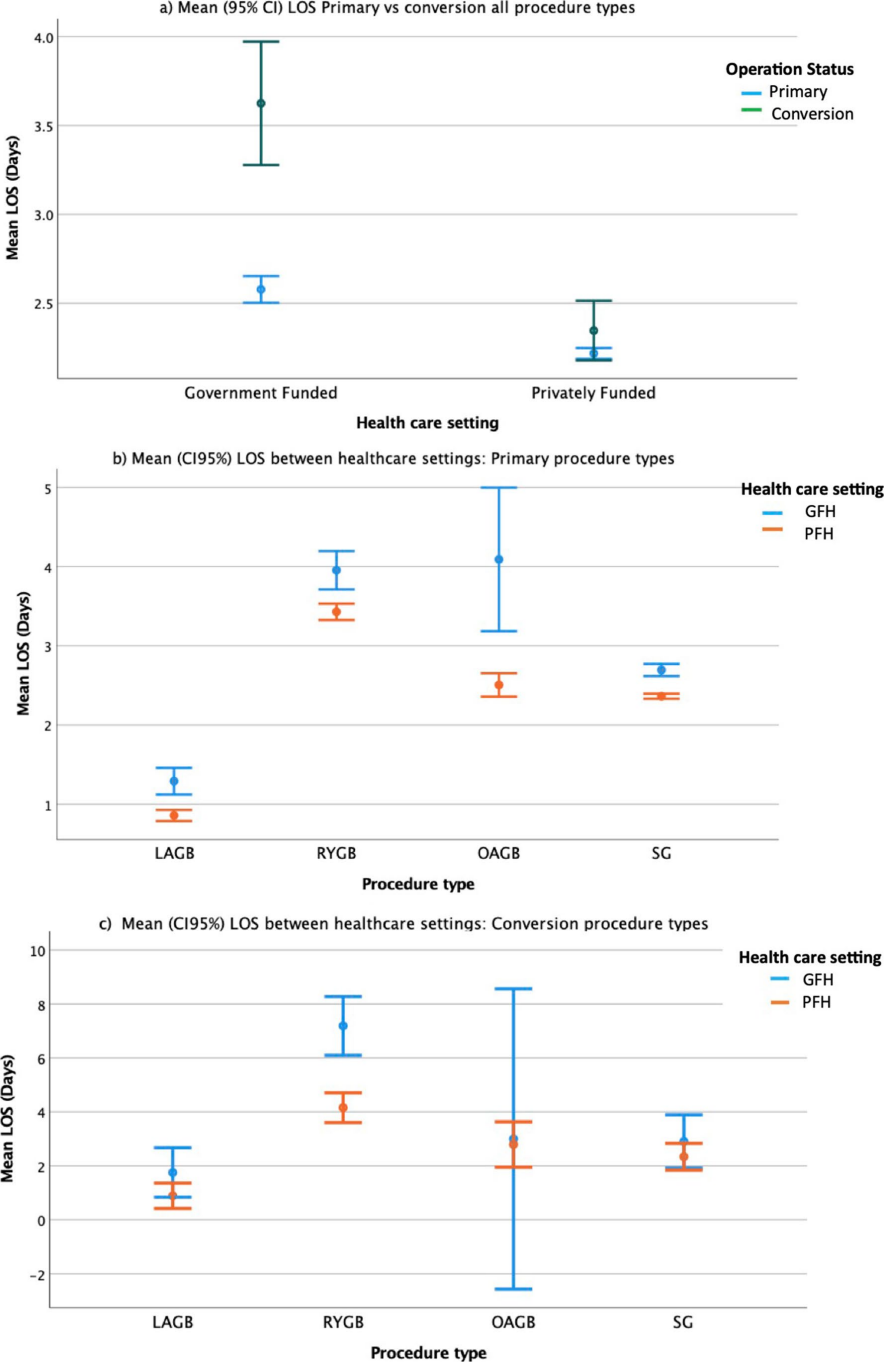
Mean (CI_95%_) LOS between healthcare settings (**** denotes *P* value < 0.001 following ANOVA)

Patients receiving primary bariatric surgery in a GFH had a mean LOS (SD) of 2.58 (2.07) days compared to 2.22 (1.58) days for individuals who attended PFH, *P* < 0.001 (Table 3). Patients who received conversion bariatric surgery in a PFH had a mean (SD) LOS of 2.35 (2.68) days compared to an average LOS of 3.63 (4.76) days for individuals who attended a GFH, *P* < 0.001 (Fig. 5a). This variance in LOS between the two hospital settings was significant when the specific procedure type was considered for primary (F = 20.90; (1.14862), *P* < 0.001) and conversion procedures (*F* = 8.86 [3.14862), *P* < 0.001) (Fig. 5b and c).

**Table 3:** Significant covariates affecting LOS, multiple linear regression

	GFH			PFH		
Covariate	Beta coefficient	95% CI	*P* Value	Beta coefficient	95% CI	*P* value
Weight at operation	0.005	0.001–0.009	0.022	0.004	0.002–0.006	< 0.001
Diabetic status (yes)	0.528	0.289–0.768	< 0.001	0.426	0.288–0.564	< 0.001
Operation status (conversion)	0.791	0.230–1.353	0.006	0.491	0.224–0.759	< 0.001
Unplanned return to theatre	4.804	3.378–6.230	< 0.001	0.778	0.111–1.445	0.022
Unplanned readmission	2.601	1.130–4.072	< 0.001	0.843	0.153–1.533	0.017
Post-operative complication (yes)	0.615	0.176–1.055	0.006	1.215	0.436–1.994	< 0.001

Covariates which significantly affected the expected LOS following bariatric surgery are presented in Table 3. Increased LOS was noted in both GFH and PFH for patients with diabetes, conversion bariatric procedures, unplanned return to theatre, unplanned readmission and if a complication was reported. Procedures performed in GFH which reported in the affirmative for these covariates had a larger increase to expected hospital stay when compared to those in performed PFH. The greatest change to expected LOS was observed in procedures which reported an unplanned return to theatre. An unplanned return to theatre following a procedure performed in a GFH extended the LOS of a patient by 4.80 days (CI_95%_ 3.37–6.23) compared to 0.77 days (CI_95%_ 0.11–1.45) in PFH, *P* = 0.022. The number of reported unplanned ICU admission was too low in either hospital setting to reliably predict impact on LOS.

## Discussion

Whilst there are many case series and randomised controlled trials confirming the efficacy of bariatric surgery, the majority of these studies have been undertaken in private hospital settings. This is one of the first studies confirming that the efficacy and safety of bariatric surgery is comparable between government- and privately funded health institutions.

In this study, participants who received bariatric surgery in GFH were older, had a higher weight and BMI and required more treatment for diabetes when compared with the PFH patients. The efficacy and durability of weight loss outcomes, as well as diabetes remission, following bariatric surgery were similar between the two healthcare settings. These positive benefits were sustained at 5 years post-operatively.

Whilst the risk of an adverse event was relatively low in both settings, GFH had a significantly higher rate of specific adverse events. This may reflect the higher risk profile of GFH patients at baseline (higher weight, older age, more complex treatment for diabetes).

In our study, patients who had a procedure in GFH had longer LOS for both primary and conversion procedures, most likely reflecting their higher base line risk and the higher frequency of conversion surgery in GFH. The covariates which had a significant impact on patient LOS were similar between GFH and PFH; however, the magnitude of effect of these covariates was greater in GFH procedures. This is in keeping with previously published work demonstrating the demographics of high healthcare consumers are mirrored in the demographics of individuals who utilise government-funded healthcare [14, 15].

All jurisdictions are challenged by the need to provide equitable access to effective treatments to all of their citizen regardless of a given individual’s ability to pay [16]. For high prevalence diseases such as obesity, that are also over-represented in the socioeconomically underprivileged; this issue is particularly challenging.

Remission of comorbidities associated with obesity following the significant and durable weight loss provided by bariatric surgery in the high healthcare consumer has been shown in case series to reduce the burden on health care systems from as early as 12 months following surgery^9^. The findings of our large data analysis confirm these findings, with positive effects sustained to 5 years post procedure. These data provide surety to government payers that there is benefit in resource allocation enhancing access to bariatric surgery.

It is possible that other initiatives which have been previously demonstrated to improve patient outcomes reduce hospital LOS, and unplanned hospital presentations/readmissions would further improve outcomes and minimise adverse events. These strategies include formal patient pre-rehabilitation programs [17], streamlined patient ward management protocols such as ERABS [18] and effective post-operative community patient care initiatives [19].

A key strength of our study is that it is representative of practice as we have utilised multicentre large data from a national quality and safety registry. By using data from Victoria rather than whole Australia, we have reduced the risk of confounders that result from the colocation of government- and privately funded health services that occurs in other states. During the study timeframe, the funding location of procedures performed in Victoria and registered with the ANZBSR was dichotomous. No privately funded bariatric cases occurred in GFH, and no government-funded procedures occurred in PFH care settings.

It must be noted that the study has limitations. The number of clinical covariates utilised in this study was restricted to the variables recorded by ANZBSR, and it is recognised that the there are other variables that could explain some of the variation in lengths of stay and especially regarding adverse events. Additionally, the reporting of defined adverse events and specific complications to the ANZBSR may have been under reported as this information was voluntarily disclosed by clinicians to the registry. Previous published studies have determined that failure to report may underestimate readmission rates by approximately 18% [20]. This study therefore provides an important to call to arms to the Australian bariatric surgical community to improve transparency by accurate reporting of adverse events to facilitate the rigorous understanding of the parameters for safety and efficiency in bariatric surgery. Although the tables reported in this paper describe the observed data well, their predictive performance would need to be investigated in multiple populations before any conclusions could be drawn on their international generalisability.

## Conclusion

Both GFH and PFH bariatric surgery in Australia provide safe and sustained weight and metabolic health improvements. There were small but statistically significant increases in reported complication rates, defined adverse events and hospital length of stay in GFH, which may relate to the significantly higher risk population GFH are treating. Government payers should be reassured that the health and safety outcomes from bariatric surgery are comparable to PFH settings and are a sound investment in community health.

## Data Availability

The data that support the findings of this study are available from the Australian and New Zealand Bariatric Surgery Registry, but restrictions apply to the availability of these data, and so are not publicly available. Data are however available from the authors upon reasonable request and with permission of the Australian and New Zealand Bariatric Surgery Registry.
